# Monitoring, evaluation, and learning: the key to building effective partnerships with government to improve maternal and child health in the Rakai and Kyotera Districts of Uganda

**DOI:** 10.3389/fpubh.2024.1188584

**Published:** 2024-08-07

**Authors:** Marc Sklar, Daniel Murokora

**Affiliations:** Babies and Mothers Alive Foundation, Kalisizo, Uganda

**Keywords:** maternal and newborn health, reproductive health, monitoring, evaluation, and learning, government-NGO partnerships, health system strengthening

## Abstract

This article emphasizes the significance of the Monitoring, Evaluation, and Learning (MEL) system within Babies and Mothers Alive (BAMA) Foundation in building effective sustainable interventions at scale. The foundation aims to enhance the availability of high-quality reproductive, maternal, and newborn care services within the government health sector. The distinguishing characteristic of the MEL system is its integration of organizational learning as a strategic approach to inform the development of dynamic program designs. To do this, it has been necessary to identify crucial requirements through open data exchange with all pertinent stakeholders. This paper demonstrates that our approach to evidence-based learning in a diverse population of locally-based actors and stakeholders, gives voice to the community-based health practitioners and patients that is necessary for transformative maternal health delivery systems. The act of sharing data has presented several possibilities for enhancing current initiatives and extending the reach and scale of our partnership model. We trace the development of the core components of learning and decision making, and reflect on the transition of the program to scale using the LADDERS paradigm. The application of our model of practice has been associated with the increased financially viability and the potential for the sustainable scaling of the program intervention.

## Organizational background and goals

Since 2004, the Babies and Mothers Alive (BAMA) Foundation has been on a journey of learning. As public health physicians, one Ugandan and one American, BAMA’s founders began with a question, how could we best improve the quality of reproductive, maternal, and newborn health systems in rural populations in Uganda. As obstetrician gynecologists, with extensive experience throughout sub-Saharan Africa, we shared a frustration with externally led interventions, characterized by top-down decision-making and driven by short-term funding cycles, that fail to generate sustainable solutions. Over the course of our long involvement in community participatory programming we have found that the ultimate experts on how to best strengthen health systems are to be found among the people using the services, who are rich in ideas, experience, and local knowledge. As a locally-led NGO, we are not just another program led by “outside experts” but rather our role is to build, gather and share knowledge, putting power and resources in the hands of those who already hold the solutions. In this paper we discuss the central role that information sharing has played in the process of engagement across multiple partners.

The 20-year journey with the communities of Rakai and Kyotera in Uganda has progressed through cycles of innovation and consolidation, with our health interventions designed to dramatically improve access to quality health care services within the government sector. The documentation of these activities, and of their successes, has enabled the organization to attract the support of government and international aid sources, creating the feasibility to undertake even more ambitious programming.

Building trust and establishing relationships, by raising awareness in local communities and by providing supportive supervision for health service staff, has also been key to building a sustainable system. In 2015, we launched our comprehensive maternal and newborn health program (MNH) as a fully integrated partnership with the Rakai and Kyotera District government health systems in the central region of Uganda.

The MNH program recognizes that the quality of health delivery services is almost entirely determined at the district government level. Within this system, while public-NGO partnerships can be a key to the implementation of effective, evidence-based health interventions, there is a need to take a longer term, more flexible approach to implementation to be able to sustainably scale to other regions. From the beginning therefore, program design has been defined by the need: for a long-term commitment, moving beyond the more traditional 2–5 year granting process; and for policies that support contextualized responses to localized influences. All of our health interventions have responded to needs identified at the community level, are in full alignment with Ministry of Health priorities, and thus our achievements have been accomplished through the collaborative efforts of BAMA staff and local partners.

**Figure 1:**
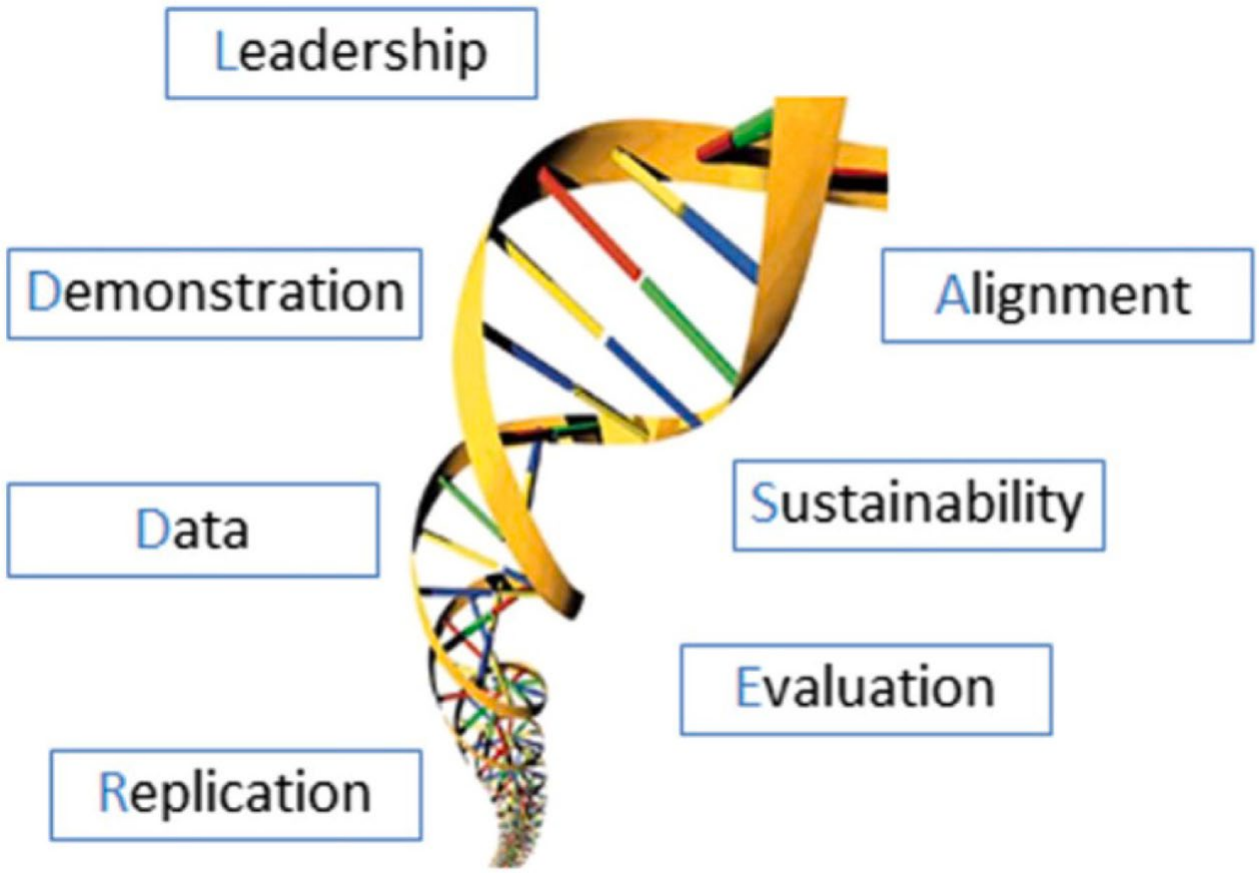
LADDERS model (used with permission).

In reflecting on our progress toward making a transition to scale we have used the LADDERS model as a guide (Figure 1). This is a dynamic paradigm for planning, implementing, and evaluating sustainable change in learning health systems (1). The acronym stands for Leadership, Alignment, Data, Demonstration, Evaluation, Replication and Sustainability. The relationships between all seven of these key components to sustainable systems change are non-linear and constantly in flux in a dynamic process. We have presented them in the order that most appropriately fits our process.

## Data

### Building an integrated data system

Deeply rooted within our communities, our staff recognized from the outset the importance of establishing systems that fostered communication and learning among all program stakeholders and beneficiaries. The collection and analysis of data, informing program design and day to day decision making was paramount.

In order to be effective, both in terms of program design, as well as our implementation strategy, the program needed access to accurate and timely data, on health outcomes, measures of access and utilization of health services, as well as qualitative data reflecting the experience of both beneficiaries and those in the health delivery system. In line with the Measurement for Change approach (2–4), building a rigorous Monitoring, Evaluation and Learning (MEL) system is foundational to the success of these partnerships, and of integrating into the government system. Sharing information and learning has also influenced the shift toward shared responsibility and shared accountability across multiple partners.

While all government facilities collect mandated health data, the quality of this data did not allow for reliable estimations of maternal and perinatal mortality. The cycle of data collection, utilization and system adjustments was also lacking. *In our first year of implementing the BAMA Program in 2016, we assessed the quality of data by measuring the* *variance between the existing data records at the health facility level, and the actual data after our rigorous assessment of data quality through cross checking of individual patient records, birth logs, operating theater records* etc. *The data reviewed covered the full spectrum of the maternal and newborn care provided in the health facilities, maternal and neonatal assessments, time and mode of delivery, among others.*

Therefore, from the outset, we committed to strengthening government health data systems and establishing methods to establish quality services through on-going learning. In 2015 we performed an extensive survey across 24, expanding to 48, district health centers, assessing the utilization and quality of reproductive, maternal, and newborn health services. Subsequent quarterly Data Quality Assessments, performed at all partnering health facilities have demonstrated dramatic improvements in data quality. Through rigorous cross checking of medical records, we have measured variance in the Health Management Information System (HMIS II) records. In 2015, only 50.3% of cases in maternal registers were accurately reported. Since 2020 we have maintained an accuracy rate of over 95%.

Data is collected electronically and synced to our integrated data warehouse, which in turn is linked the government’s Health Management Information System (HMIS II). Our data collection and analysis process begins at the health facility level, where BAMA trained health center and hospital staff routinely record client information in paper-based medical records and clinical logbooks. BAMA staff perform data quality assessments, collecting and validating this data on a regular basis using laptops, mobile phones, and tablets. Data collected by field and district health staff is regularly crosschecked for accuracy and evaluated for data variance at the facility and program level. All project data collected is analyzed routinely using statistical analysis and visualization packages, including Stata 13, Power Bi and MS Excel. We use the Atlas.ti software program to locate, code, and annotate qualitative data.

The creation of an integrated data warehouse requires a long-term commitment from, and to, facility level staff. Single step training is insufficient, and on-going mentoring to create joint evaluation of data quality has been crucial. These activities are both time consuming and labor intensive, a factor not always reflected as a priority in program funding. BAMA has been fortunate to have received unrestricted funding from its US-based partner affording the freedom to invest the necessary capacity building systems to continue to generate high quality health data systems. Our integrated data system allows regular analysis of data in real time and the creation of infographics, allowing clear dissemination of our program outputs and impacts to Ministry of Health, District Health Teams, stakeholders, and our funders. *Sharing our progress in improved health outcomes at multiple national forums and Ministry of Health Technical Working Groups has promoted the dissemination and adoption of key innovations in the BAMA Program model. For example, our Maternal Newborn Obstetric Complication Survey or MNOCS, which BAMA designed and implemented, has now been integrated into the national Health Management Information System (HMIS), supporting the measurement of Case Fatality Rates, which are then used to inform decision making and where to invest time and resources to improve quality of care.*

We have also utilized focus groups and interviews to systematically engage health workers, beneficiaries, community members and partners in formative research through on-going discussions on an individual and group level. The BAMA staff who facilitate this dialog are counselors, midwives, physicians, and social workers, who are grounded in relationships of service and trust. Utilizing a Rapid Cycle Learning approach, we are able to remain flexible and respond to changing conditions in the field.

## Demonstration

### Using information to build trust

Beginning with very modest resources, the information system has built up information on progress. It has tracked the achievement dramatic improvements in health outcomes. From 2015 through to the present we have seen (5):

- 74% decrease in maternal deaths at 48 partnering health centers and hospitals.
- 44% decrease in perinatal deaths at 48 partnering health centers and hospitals.
- Case Fatality Rate (CFR; the percentage of women dying from major obstetric complications) was reduced from 1.7% to 0.3%.
- 66% reduction in complicated abortions.
- 70% reduction in life-threatening hemorrhage both during pregnancy and postpartum.
- 38% reduction in obstructed labor.
- Reduction in the Decision to Delivery interval (DDI) for the performance of caesarian interventions from 124 min to under 40 min.
- Increase in facility deliveries from 9,522 to 16,071 per year.
- 27,373 women transported from village to health center and 3,229 women receiving emergency referral transport.
- Time for referral has been reduced from 3 ½ h to just 34 min since June 2021.
- 2,240 newborns admitted to our three recently constructed NICUs, in 2019 92.3% were discharged alive. In 2023 the survival rate increased to 97%.

The data system used, tracking all major life threatening complications through the BAMA-designed Maternal, Newborn, Obstetric Complication Survey (MNOCS), has provided detailed evidence of progress and success. This approach also now been integrated into the health data systems by the national Ministry of Health. The shared ownership of the health system, with its integrated information and learning process, has built trust in the BAMA-district government partnership and strengthened our advocacy at the Ministry of Health for increased investment, as well as for adoption of innovations in the health system.

The BAMA Maternal and Newborn Health Program is designed around the 3-Delays model (6). The vast majority of preventable maternal and newborn deaths in limited-resource settings can be linked to one or more of these 3-delays.

- First Delay: The delay in the decision to seek skilled maternal and newborn care.
- Second Delay: The delay in reaching skilled maternity and newborn care once the decision is made.
- Third Delay: The delay to receive quality care once reaching a health facility.

The health data collected by BAMA staff and government Health Information Officers informs decision making in identifying and addressing barriers to obstetric care in our resource limited communities (7). Our data has enabled us to monitor and respond to all three delays by increasing demand for and utilization of antenatal care, institutional obstetric and newborn care, barriers to reaching care due to limited transportation options, and the quality of care provided at government-funded health centers and hospitals.

This model, and reductions observed in indicators such as institutional maternal and perinatal mortality, and case fatality rates, illustrate the pathway to the achievement of dramatic improvements in access to quality care, supporting our theory of change that increasing skilled attendance saves lives. The skills we have fostered in health facility staff, however, go beyond direct health care to include the effective use of data systems.

Improved skills in reporting and analysis enables local health leaders to design a rapid response to health delivery bottlenecks.

## Alignment

### Re-design in response to emerging evidence

From its initiation, the BAMA Program was designed to partner with local communities and district health systems to address the 1st and 3rd delays. The Mama Rescue Project is a transportation initiative, nested within the comprehensive BAMA Program to address the 2nd delay, barriers to skilled attendance at delivery due to lack of access to transportation from village to health facility. It uses a simple mobile phone app, linking women in labor to motorcycle taxi drivers. It also connects women in need of emergency referral to automobile taxis. We have transported over 27,000 women in just 2 years, while reducing referral times from 3 1/2 h to just 34 min. Limited resources had initially prevented us from addressing barriers to transportation. In 2017, a small proof of concept was performed in the Kasese District of Uganda to evaluate this innovative IT solution. A close evaluation of the pilot led to many valuable lessons.

While BAMA was involved in the initial design, Mama Rescue was launched as an independent stand-alone project and ultimately suffered from a lack of full partnership with the district health system. By nesting Mama Rescue within a comprehensive maternal health partnership with government, implemented by an organization with deep roots in the communities served, BAMA was able to leverage years of trust to build a broad base of community support.

Mama Rescue now facilitates rapid referral of women with life threatening complications of pregnancy. Additionally, this application allows health center midwives to enter vital clinical information that ensures that referral hospital staff are maximally prepared and delays in comprehensive emergency obstetric and newborn care are reduced. We are able to track referral times and appropriately respond to delays in a timely fashion.

From the onset of the BAMA Program, our needs assessment took note of the high rate of adolescent pregnancy (births per 1,000 women age 15–19), 21% in our districts and the lack of adolescent-friendly maternal and reproductive health services in Rakai and Kyotera. In response, the Mama Ambassador Program (MAP) was initiated, employing monthly peer support parenting groups to improve early childhood development and mental health for adolescent mothers. As with all BAMA Program innovations, we gathered data. To support an evaluation of proof of concept we measured child development and maternal mental health. As the data was collected it became clear that over 20% of young mothers recruited into the program reported themselves as survivors of sexual and gender-based violence (SGBV). This then became a major focus of our expanded MAP, with the hiring of a full time SGBV counselor, a clinical social worker, and engagement with district and legal authorities to improve survivor support services.

The original MAP was centralized with peer support groups occurring at two district hospitals. Feedback from our local partners, as well as the mothers themselves, revealed that limiting our support groups to the district hospitals created barriers to access. As we scaled the MAP, we expanded services to 10 health centers, bringing the groups closer to our beneficiaries and extended families. Data collected on the prevalence of SGBV, as well as forced marriage, guided our program design decisions as we scaled the MAP, expanding male engagement, as well as outreach to extended family, civil society, and the legal system.

As we are in constant communication with these close partners, we receive regular feedback on program implementation, as well as the evolving needs of our beneficiary communities. This was especially critical during the lockdown phases of the COVID pandemic, where we were forced to make dramatic changes in our Mama Ambassador Program, rapidly shifting from a peer-support group to home visit model for over 400 adolescent mothers and babies. [See: our video SMILING THROUGH THE STORM: FINDING SILVER LININGS IN THE MIDST OF THE PANDEMIC (8)].

Using data for program design and decision making has shaped other projects within our comprehensive BAMA Program. The My Pads Program was launched in 2011, as an after-school sexual and reproductive health (SRH) educational program for adolescent girls and young women, with a focus on menstrual health and hygiene. Our data had already directed us to gaps in our existing model, where at risk out of school girls and boys were being ignored. Discussions with school and political leadership also exposed the limitations in delivering a comprehensive SRH educational program in a primary school setting. This has led to the re-design of My Pads into a more holistic three-tiered model, with both in-school and out of school peer groups targeting both adolescent women and men, linking them to youth-friendly SRH services at partner health facilities.

The importance of leveraging the strength of the government health system through trusted relationships with both government and community partners has been a key lesson in the journey toward sustainability. *What distinguishes the BAMA partnership model is the full integration of BAMA program interventions into the government health delivery system. BAMA’s core implementors are not our own staff, but rather a highly motivated corps of BAMA-trained government employed Mentor Midwives and Physicians and Community Health Workers who are empowered as Mama and Papa Ambassadors. Initially, District Health Teams and health providers were skeptical and defensive, as they confronted the reality of major gaps in access to quality of care that led to high levels of maternal and newborn deaths. Ultimately, it has been the trust built over years, and the fact that we work as colleagues rather than outside experts that has led to our successes to date. What is required to build this trust is the willingness to commit long term to our government partners, not limited by grant funding cycles, and to engage fully in a learning journey with the partners and beneficiaries who ultimately are the source our success.*

## Leadership

### Learning with our partners

Our Monitoring and Evaluation staff is comprised of five local data scientists. In sharing the messages they produce they are joined by a corps of faith-based, civil society and cultural leaders as champions for reproductive health and rights. Backed by local evidence, these champions support the outreach and communication with our beneficiary communities.

All of our program interventions involve dynamic community outreach, affording our staff ample opportunities to engage with women, adolescents, extended families and community leaders in an active and on-going dialog regarding their needs and the most effective means to address them. Over the past 8 years this outreach network has further been supported by 150 government Community Health Workers who have been trained as Mama and Papa Ambassadors. Working at the village level these ambassadors are personally connected to thousands of families and serve as a conduit for key information facilitating various interventions such as counseling and referral.

Over the past 8 years, since we launched our health partnership with district government and the Ministry of Health, we have continuously engaged our partners in knowledge sharing which informs program design and implementation. We participate in monthly Ministry of Health (MoH) Technical Working Groups through which we engage key Reproductive, Maternal, Newborn, Child, and Adolescent Health (RMNCAH) stakeholders. We present our projects at inception and receive feedback on priorities, approaches and the results frameworks. This is followed by monthly performance reviews and progress reports to the MoH.

The district level biostatisticians are part of the team. We conduct onsite, health facility feedback meetings as well as similar meetings at district level. Feedback from these engagements directly inform course changes in our implementation strategy to support maximum impact.

## Evaluation

### How has MEL informed our transition to scale strategy?

Since 2015, we have learned that a trusted NGO-District government partnership can support transformative change in the access to, and quality of, reproductive, maternal, and newborn health services, serving the most vulnerable populations in Uganda. Core to our success has been our longstanding relationships to district governments and the rural communities we serve, but this reliance on deeper, locally focused relationships comes with resource/funding challenges. Government and institutional funders often seek larger organizations with national or international scope but without the relationships and contextual knowledge that has contributed to our success. We have come to realize that scaling the BAMA model will require a pathway built on expanded partnerships with government and other NGOs that share our mission and vision, supporting the integration of key components of program interventions.

## Sustainability

### The proposed transition to scale strategy

The three corner stones are seen to be:

- Expand the BAMA Program to our neighboring districts in the Masaka Region: Our initial goal (3-years) is to expand to the two adjoining districts of Masaka and Masaka City, followed by scaling to the entire Masaka Region of nine districts serving a total of 2,218,286 people. Directly overseeing this expanded BAMA Program implementation will allow us to adapt our model to support sustainable integration of our core interventions into the existing government health system, our ultimate goal.
- Identify potential NGO partners in Uganda working in reproductive, maternal, newborn, and child and adolescent health (RMNCAH) and development: Babies and Mothers Alive has a long history of high impact partnerships with national and international NGOs who are aligned with our mission. The establishment of a network of RMNCAH NGOs will support the dissemination our BAMA core innovations, and extend and expand the reach of our program. Through such partnerships, and the establishment of an RMNCAH Stakeholder network, we will support NGO-government collaboration, promoting BAMA integration into the government health system.
- Integrate BAMA and its data systems into the district and national health system: In building the BAMA Program in full partnership with district government and the Ministry of Health, we participate in multiple forums at the national level overseeing maternal, newborn, reproductive, and adolescent health. Our senior staff are respected thought leaders. We”ll leverage our success to date, encouraging the adoption of key components of our program model. For example, we have already seen our Maternal and Newborn Obstetrics Complication Survey (MNOCS) integrated into the government health data system.

## Replicability

### Taking context into account

In the next stage of our innovation process, it is evidence from our four currently partnering districts that will guide the initial design of new adopting districts. However, as they will represent a diversity of contexts and demographics, results in these second-stage districts will need to be reviewed to ensure the program remains fit for purpose.

This cycle of design, review and re-design will be carried over to set the stage for a third-phase of Uganda wide spread of those interventions that have demonstrated sufficiently compelling cost benefit justification and a robust scaling approach.

## Discussion and conclusion

Working to be catalyst for transformational change requires a dynamic learning process that is on-going, and not static. BAMA’s partnership model is built on fostering a learning environment with our many stakeholders and the families we serve.

Investing resources, both financial and human, in our MEL process has greatly impacted the design, implementation strategy, and ultimately, the success of our comprehensive program interventions. It has also strengthened our relationships with partners and stakeholders, providing an organizational structure that promotes on-going communication and trust. Our partnership model is built around this learning process, a model acknowledged as integral by other implementing organizations (9–11).

We have experienced challenges in our MEL journey over the past 8 years that have placed limitations on our progress. Like many locally based NGOs working in limited resource settings, our constraints in funding define the time available for full engagement in monitoring, evaluation, and learning. Often, we find our staff and partners struggle to meet the ambitious program targets and benchmarks that we establish in order to address critical gaps in health care delivery negatively impacting our communities. We continue to engage with our donors, both governmental and institutional, to broaden their funding for these activities which have proven so vital for successful design and implementation of our programs. We also see the value of strengthening our qualitative data process so that we can more formally collect and analyze this essential learning. Finally, our focus on cost effective program implementation has limited our efforts in designing research studies that can definitively prove our model’s effectiveness. Instead, we have focused on building systems that builds collective use of data to improve responses to need.

Use of a reflective framework that the LADDERS paradigm provides has guided our focus on what is happening and what is not happening. The biggest limitation to the design of successful work planning is investing adequate time for review and reflection. We hope to be more successful in the future in making this a priority, especially regarding nurturing our staff and partners. In this process, the intentional use of MEL systems plays a major role. With respect to Leadership and Alignment, we have yet to design clear indicators that assess the integration of our program interventions into the government system. Adequate investment of time and resources in our on-going engagement with government partners will be crucial to developing these indicators that will define the success of the Replication and Sustainability of the BAMA Program as we scale to new districts. Always acknowledging what we have yet to learn has kept us open to new discoveries and the willingness to test novel approaches to achieve our shared goal of quality health care for the women and children of our communities.

## Data availability statement

The raw data supporting the conclusions of this article will be made available by the authors, without undue reservation.

## Author contributions

All authors listed have made a substantial, direct, and intellectual contribution to the work and approved it for publication.
